# Improving the Lifetime of CsPbBr_3_ Perovskite in Water Using Self-Healing and Transparent Elastic Polymer Matrix

**DOI:** 10.3389/fchem.2020.00766

**Published:** 2020-10-06

**Authors:** Livy Laysandra, Yong Jie Fan, Cecilia Adena, Yen-Ting Lee, Ai-Nhan Au-Duong, Liang-Yih Chen, Yu-Cheng Chiu

**Affiliations:** ^1^Department of Chemical Engineering, National Taiwan University of Science and Technology, Taipei, Taiwan; ^2^Graduate Institute of Applied Science and Technology, National Taiwan University of Science and Technology, Taipei, Taiwan

**Keywords:** random copolymer, stretchable, self-healing, Inorganic halide perovskite, butyl acrylate, N-(hydroxymethyl)acrylamide

## Abstract

This study developed a simple and efficient strategy to stabilize inorganic halide perovskite CsPbX_3_ at high relative humidity by embedding it into the matrix with elastic and self-healing features. The polymer matrix has a naturally hydrophobic characteristic of *n*-butyl acrylate segment (*n*-BA) and cross-linkable and healable moiety from *N*-(hydroxymethyl) acrylamide segment (NMA). It was chosen due to the provisions of both a surrounding protective layer for inorganic perovskite and elastic, as well as healing ability to the whole organic-inorganic composite. This fabricated CsPbBr_3_/PBA-*co*-PNMA composite was demonstrated to stably persist against the suffering from hydrolysis of perovskites when exposed to a high moisture environment. The PL intensity of the composite after crosslinking was found to be relatively stable after 30 days of exposure to air. Upon water immersion, the PL intensity of composite only showed a decrease of 32% after the first 6 h, then remained stable for 6 h afterward. Furthermore, this fabricated composite was not only flexible and relatively transparent but also exhibited excellent self-healing capability in ambient conditions (*T* = 25°C), in which the self-healing efficiency after 24 h was above 40%. The tensile strength and stretching ability of 5 wt% perovskite content in the random copolymer were observed to be 3.8 MPa and 553.5% respectively. Overall, flexible and self-healing properties combining with high luminescence characteristics are very promising materials for next-generation soft optical devices.

## Introduction

In recent years, there has been focused and rapid progress in the synthesis of all-inorganic perovskite quantum dots of cesium–lead–halide (CsPbX_3_, where X = Cl, Br, I, or their binary mixture) materials with high performance for solar cells, LEDs, lasers, and photodetectors (Nedelcu et al., 2015; Protesescu et al., 2015; Li X. et al., 2016; Makarov et al., 2016; Seth and Samanta, 2016). CsPbX_3_ are well-known for having high defect tolerance (Kang and Wang, 2017), size- and composition-tunable emission wavelength (Tong et al., 2016; Zhu et al., 2017), narrow emission bandwidth (Ravi et al., 2016), easy processability, and a high photoluminescence quantum yield (PLQY) (Wei et al., 2016). More importantly, inorganic CsPbX_3_ exhibits higher thermal stability compared with hybrid organic-inorganic halides (CH_3_NH_3_PbX_3_, X = Cl, Br, I) (Cells et al., 2015). However, under humid conditions or contact with water, inorganic halide perovskite suffers from hydrolysis owing poor its chemical stability, which leads to ion-exchange reactions and decomposition through inevitable fast anion exchange, and hence causes emission band broadening and emission color instability (Habisreutinger et al., 2014; Shi et al., 2017).

Various methods have been studied to avoid the oxidation and the degradation of CsPbX_3_ caused by sensitivity to moisture and air, including protective coating, cross-linking of ligands, and polymer blends. For example, Gomez et al. prepared encapsulation of colloidal cesium lead halide perovskite NCs within solid lipid nanoparticles (SLNs) showing an improvement in the stability of the PeQDs colloidal encapsulation underwater, arresting its composition homogenization for at least 2 months (Gomez et al., 2017). However, SLNs have poor mechanical strength, lack of flexibility, and are susceptible to cracking, which are obstacles to developing applications for optoelectronic devices. Palazon et al. have demonstrated that low flux X-ray irradiation, which induces the formation of C=C bonds between the organic ligands coated on the QD surface, could greatly enhance the stability of cesium lead halide NC films up to several weeks in air (room temperature, ambient humidity), 1 day in Milli-Q water, or 3 days in the biological buffer (PBS 1 ×) (Palazon et al., 2016). However, these films may not be suitable for electronic applications due to their high organic content and instability in humid conditions.

To date, blending CsPbX_3_ with polymers has presented a simple, cost-effective, and promising method to improve its stability with functional properties. For example, a previous study focused on embedding CsPbX_3_ in polymer matrices such as poly(methyl methacrylate), poly(butyl methacrylate), or polystyrene (Xin et al., 2018), which could retain quantum yield in air and water for 1 month. As we know, the matrices of PMMA and PS, as rigid and non-healable materials, may narrow their utilization under typical mechanical strain, while PBMA provides flexible properties with low tensile stress (1.1 MPa) that are thus able to stretch up to 170%. However, there is no traceable healing ability in PBMA. Although significant progress has been made in CsPbX_3_ preparation, major challenges remain in finding practical applications for solid-state CsPbX_3_.

Self-healing is a process in materials that possess the ability to automatically repair their structure and restore their original state, with full or partial recovery of mechanical strength after damage, and can prolong the lifetime and reduce the maintenance costs (Li C. H. et al., 2016). Over the last 20 years, researchers have realized that the accumulation of undesirable material damages can occur at any time due to changes in volume, abrasion, micro-cracks, operational fatigue, and degradation over time (Chen et al., 2017). Taking this into account and inspired by the autonomous healing ability of human skin, scientific, and industrial research has in recent years experimented with introducing self-healing materials into a polymer system as a way to improve the performance and safety of electronic devices. Up until now, a large number of self-healing materials have been developed and applied in coatings (Zhang et al., 2017; Ataei et al., 2019), sensors (Lei et al., 2017; Liu et al., 2018), solar cells (Zhao et al., 2016; Jiang et al., 2019), supercapacitors (Lv et al., 2018; Liao et al., 2019), and e-skins (Someya and Amagai, 2019). Using a large variety of different healing mechanisms, self-healing through H-bonds is an area of interest in this research. The typical bonding energies of H-bonding are usually in the range of 5–30 kJ mol^−1^ (West, 2018), which is much weaker than covalent bonds (≈377 kJ mol^−1^ for C–C bonds) (Zhang et al., 2018). Despite being ≈10 times weaker, H-bonding greatly affects the bulk viscoelastic properties of polymers, the degree of crystallinity, and phase separation. For example, Yanagisawa et al. have demonstrated mechanically robust polymers, via tailored noncovalent crosslinks by H-bonds derived from thiourea, that heal upon gentle compression. The amorphous materials have low-molecular-weight polymers that allow substantial segmental motions, and have a large number of less ordered hydrogen bonds (Yanagisawa et al., 2018). Their healing properties are dominated by segmental movements such as the exchange of H-bonded thiourea pairs, leading to the interpenetration of polymer chains at the fractured part.

Adopting a different approach to previous studies, we utilized a novel amorphous poly(butyl acrylate-*co*-*N*-(hydroxymethyl)acrylamide) (PBA*-co-*PNMA) as a matrix to incorporate inorganic halide perovskite CsPbX_3_, where the introduced butyl acrylate (BA) has been served as a soft segment in numerous potential applications (Wen et al., 2017; Hsieh et al., 2018). In our work, PBA was chosen not only because of its softness with a low glass transition temperature of about −54°C, but also due to its hydrophobic nature which can constructs barriers between water and inorganic halide perovskite. PNMA, as a thermal-triggered self-crosslinker, is also proposed as a way to avoid poor elastic properties by forming covalent and dynamic H-bonds between NMA-segments through chemical and physical self-crosslinking reaction. The contribution of the covalent cross-link enables elasticity to be controlled, and leads to improved mechanical toughness, while dynamic H-bonds trigger an ability to self-heal at room temperature. Thus, strategy for fabricating soft, self-crosslinkable, self-healing, and even water-proof features of luminescent organic-inorganic composite are achieved by integrating inorganic halide perovskite CsPbX_3_ into PBA*-co-*PNMA polymer.

## Experiment

### Reagents and Materials

All reagents were used as received from commercial suppliers without further purification unless otherwise specified. Butyl acrylate (BA) and N-(hydroxymethyl)acrylamide (NMA) were purchased from Tokyo Chemical Industry Co., Japan, and Butyl acrylate was purified by passing an aluminum oxide column before use. 2,2′-Azobis(2-methylpropionitrile) (AIBN) as an initiator, supplied by Sigma-Aldrich. Halide Perovskite CsPbBr_3_ was received from Prof. Liang-Yih Chen using a similar procedure as reported in previous work (Nedelcu et al., 2015).

### Preparation PBA-*co*-PNMA as a Random Copolymer

A one-step free radical polymerization reaction was carried out under argon condition, unless noted. A 200 mL two-neck schlenk flask fitted with a magnetic stirrer and covered with a rubber septum was charged with NMA (2.83 g, 28 mmol) and AIBN (45.98 mg, 0.28 mmol) in a vacuum system. Periodically, the air in the two-neck schlenk flask was removed by changing the vacuum system to the argon condition three times. BA (6.1 mL, 42 mmol) and 32 mL methanol were mixed into a round bottom flask under argon condition for 30 min at room temperature. The liquid mixture in the bottom flask was moved to the two-neck round bottom flask containing solid materials. The reaction was placed into a preheated oil bath at 60°C and stirred at 320 rpm for 24 h. The unreacted monomer in the resulting colorless solution was removed using a dialysis membrane and then concentrated by a rotary evaporator at low temperature. The values for ^1^H NMR (DMSO_d_6_, 600 MHz) δ (ppm): 0.76–1.06 (**3H**, –OCH_2_CH_2_CH_2_CH_3_), 1.25–1.40 (**2H**, –OCH_2_CH_2_CH_2_CH_3_), 1.44–1.75 (**2H**, –OCH_2_CH_2_CH_2_CH_3_), 3.82–4.17 (**2H**, –OCH_2_CH_2_CH_2_CH_3_), 4.27–4.93 (**2H**, –CONHCH_2_-), 5.4–5.76 (**1H**, –NHCH_2_OH), 7.95–8.74 (**1H**, –CONHCH_2_).

### CsPbBr_3_/PBA-*co*-PNMA Composite Design

**Scheme 1:**
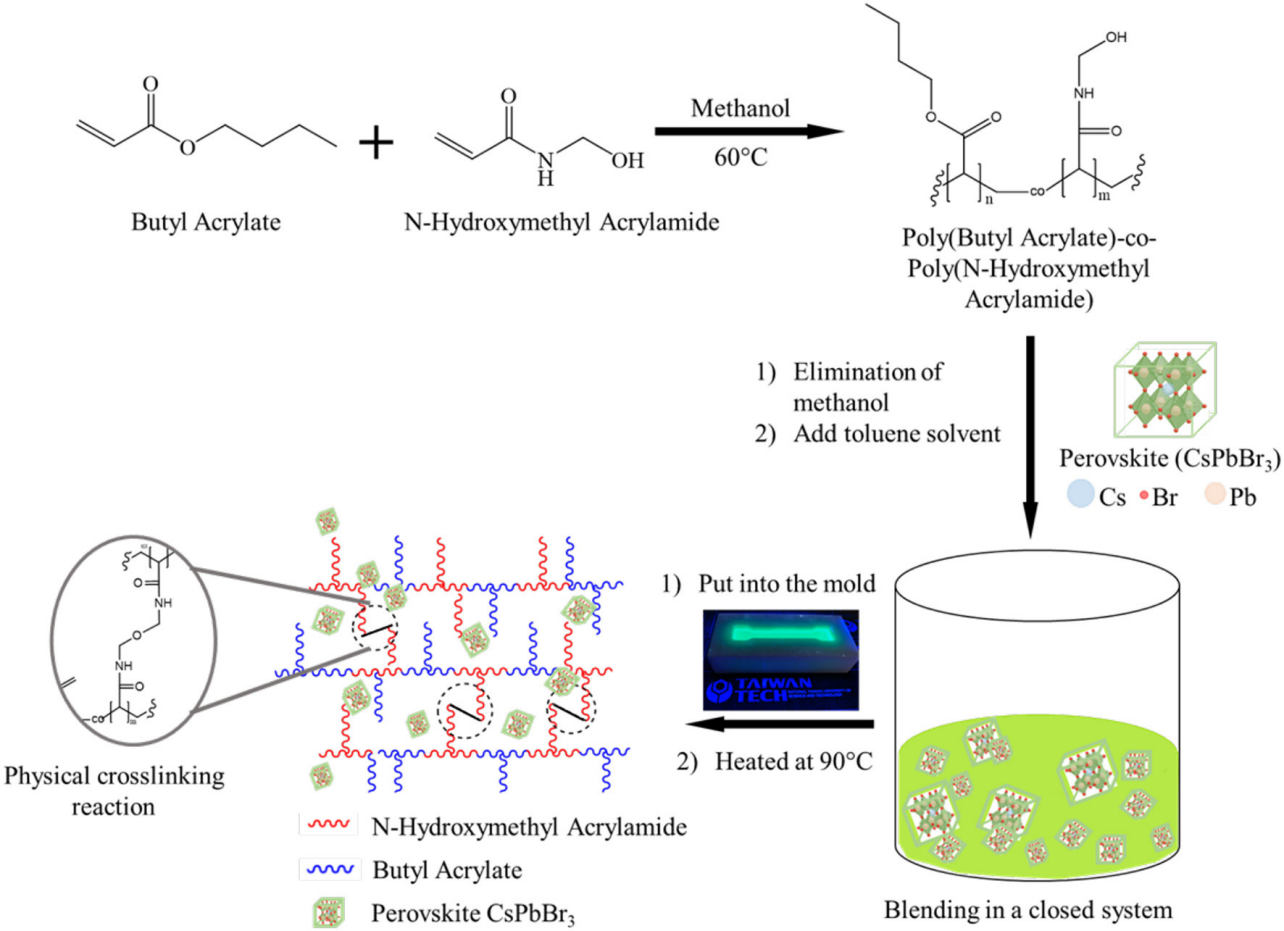
Illustration of the preparation and structure of composite as organic-inorganic CsPbBr_3_/PBA-*co*-PNMA material.

The synthesis procedure for preparing 1 and 5 wt% of CsPbBr_3_/PBA-*co*-PNMA composite was presented in Scheme 1. At first, methanol as the solvent of pristine PBA-*co*-PNMA was removed using a rotary evaporator and replaced by toluene. This replacement of methanol with toluene is needed because the prepared CsPbBr_3_ is sensitive to the methanol. To prepare 1 wt% of CsPbBr_3_ in polymer, 1 mL of PBA-*co*-PNMA (with solid content 140 mg/mL) was taken and blended with 0.14 mL of CsPbBr_3_ (10 mg/mL in toluene) under magnetic stirring at room temperature for 2 days to obtain homogenous mixture. Furthermore, 0.7 mL of CsPbBr_3_ was also blended with 1 mL of PBA-*co*-PNMA to prepare 5%wt CsPbBr_3_/PBA-*co*-PNMA. The blending process took longer due to the high-molecular-weight of the copolymer.

### Characterization

To confirm the successful PBA-*co*-PNMA synthesis and determine the conversion reaction, the copolymer was analyzed by ^1^H-nuclear magnetic resonance (NMR) spectra using a 600 MHz on a Bruker Avance III HD-600 spectrometer with DMSO_d6 as solvent at 20°C. The Gel Permeation Chromatography (GPC) analysis performed at 40°C in tetrahydrofuran as eluent (flow rate, 1.0 mL min^−1^). Thermogravimetric analysis (TGA) was performed using Q50 V20.13 TGA under the following conditions: a heating rate of 10°C/min, a temperature range of 30–700°C, sample weight range of 5–10 mg, and nitrogen flow at 60 mL/min. Differential scanning calorimetry (DSC) was performed using a DSC2-00573 (192.168.0.5) with a heating and cooling rate of 10°C/min from −80 to 110°C in a nitrogen gas environment. To investigate the interaction between CsPbBr_3_ and copolymer, PBA-*co*-PNMA with/without perovskite was analyzed by Fourier transform infrared (FTIR) on Spectrum Two (Perkin Elmer) with wavenumbers ranging from 4,000–400 cm^−1^. The phase structure and the d-spacing CsPbBr_3_ were characterized by Grazing incidence X-ray diffraction (GIXRD) patterns, and the size distribution of CsPbBr_3_ was analyzed by Transmission Electron Microscopy (TEM). All PL measurements including liquid and film forms were performed at room temperature using a commercial spectrometer (Horiba JobinYvon Nanolog-3 spectrofluorometer) equipped with an InGaAs NIR detector and a 20 nm bandpass for both emission and excitation. The PL spectra obtained were scaled according to the measured excitation power. The absorption spectra of pristine perovskite were recorded with a JASCO V676 absorbance UV-Vis spectrophotometer with a matched pair of 1 cm path length quartz cuvettes. The corresponding solvents were used as a baseline reference for every spectral measurement.

### Mechanical, Self-Healing, and Cycle Tests

The preparation of random copolymer or CsPbBr_3_/PBA-*co*-PNMA composites was conducted by similar procedures. Firstly, the sample was poured into a dog-bone shaped mold with a width of 7 mm, a thickness of ≈ 1 mm, a Gauge length of 25 mm; secondly, the sample was left for 3 days in an open space to reduce the amount of solvent in the sample. Lastly, the dried sample was put into an oven at 90°C to allow a crosslinking reaction at a certain time. Tensile properties and self-healing efficiency were measured using a universal testing machine Shimadzu – EZ-EX and were recorded with Trapezium X software. The strain rate of 50 mm/min was adopted under ambient conditions. There are many methods to determine the healing efficiency. Essentially, the standard self-healing procedure involved: (1) measuring some original material properties; (2) introducing damage into the material by cut into half by a razor blade; (3) allowing the healing process to occur by connected each fresh fracture surface; and (4) measuring the healed material and comparing it to the original property. It is important to note that there was no additional external force required for connecting the broken parts in this experiment. The self-healing abilities of the copolymer and composite film in this research were calculated through the following equation:

(1) $$\begin{array}{l} \varepsilon =\;\frac{K_{IChealed}}{K_{ICoriginal}} \end{array}$$

Where ε is defined as the healing efficiency of the sample to recover fracture toughness, K_ICoriginal_ is the fracture toughness of the original sample, and K_IChealed_ is the fracture toughness of the healed sample. All of the mechanical and self-healing tests were repeated three times.

To further verify the recoverability and self-healing of the copolymer and composites that rely on covalent and reversible H-bonds included in the NMA chains, cyclic tensile tests were carried out at a constant loading-unloading rate of 50 mm min^−1^ in different large strains of 100 and 300%. The repeatable cycle test for each different-large strain is two times with a relax time for each cycle of 5 min. First, specimens without any special treatment were loaded-unloaded to achieve 100 and 300% extensions. After that, the specimens were left to return to their original sizes. The cycle tested specimens were cut into two pieces and the cut surfaces were put in contact and then stored for 1 day. The healed-specimens were pulled to the same extensions as in the initial cycle test. The cycle tests were then conducted to understand the nature of covalent and reversible H-bonds since it is directly related to the ability of the copolymer system to recover and self-heal.

## Results and Discussion

**Figure 1:**
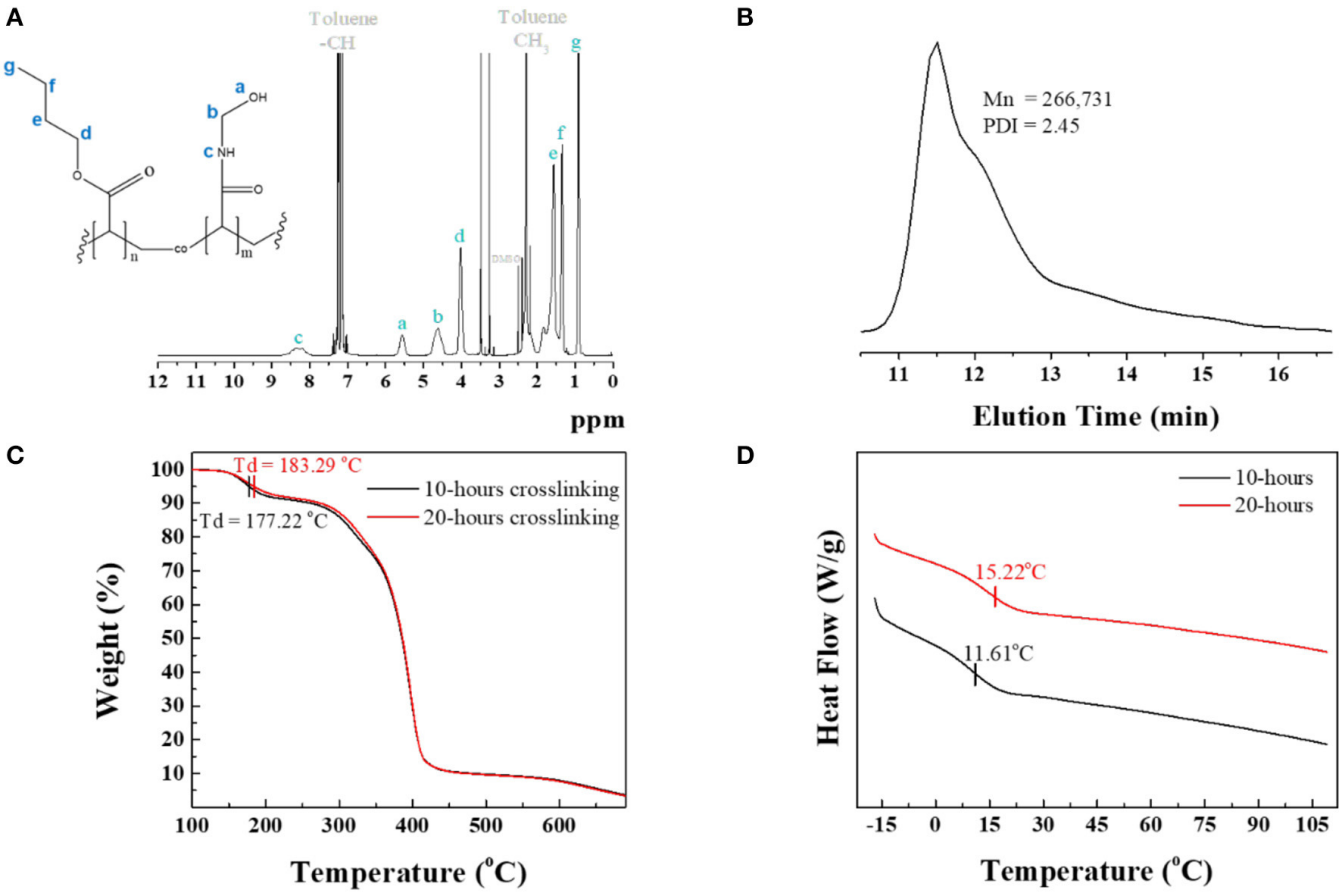
**(A)** ^1^H NMR spectrum analysis of PBA-*co*-PNMA in toluene as solvent. **(B)** GPC analysis result. **(C)** TGA curves measured in the temperature range of 100–700°C at a heating rate of 10°C/min. **(D)** DSC curves in the 2nd heating cycle of DSC analysis for 10 and 20-h crosslinking of PBA-*co*-PNMA.

The corresponding ^1^H NMR spectrum in Figure 1A shows the protons characteristic of both BA and NMA segments and reveals the monomers composition from relative integration using each segment peak, at 0.76–1.06 ppm (–CH_2_CH_2_CH_3_) of BA and 7.95–8.74 ppm (=CHNHCH_2_) of NMA. The ^1^H NMR spectrum showed that the BA:NMA molar ratio for PBA-co-PNMA was 6:4, which is identical to the feed molar ratio of the BA and NMA segments. The Mn, Mw, and polydispersity (PDI) values of PBA-*co*-PNMA were found to be 109 kDa, 267 kDa, and 2.45 respectively, estimated from Gel Permeation Chromatography (GPC) measurement (Figure 1B).

Thermogravimetric analysis (TGA) was conducted to investigate the thermal decomposition temperature (*Td*) of the copolymer PBA-*co*-PNMA in various crosslinking time conditions. Figure 1C indicates that the *Td* of the copolymer was increased by the increment of crosslinking time because longer crosslinking time establishes more covalent bonds in-between NMA segments, which also increases the solidity of the polymer. A polymer with a 20-h crosslinking time has higher *Td* at 183.29°C compared with a polymer with a 10-h crosslinking time. The weight of this polymer decreases dramatically at 270–420°C.

Differential scanning calorimetry (DSC) was utilized to determine the glass transition temperature (*Tg*) of PBA-*co-*PNMA with different crosslinking time. Figure 1D shows that a copolymer with shorter crosslinking time (10 h) has a lower *Tg* at 11.61°C compared with a copolymer with 20-h crosslinking. The glass transition temperature difference can be affected by the number of the hydrogen bonding of copolymers. Longer crosslinking time can decrease the hydrogen bonding interaction in the polymer and restrict the distance between copolymer chains, reducing the mobility leads and further restricting the copolymer chain movement, leading to Tg enhancement. Both *Tg* of the copolymers were below room temperature, which indicates that the copolymers can stay in a rubber-like state at room temperature.

**Figure 2:**
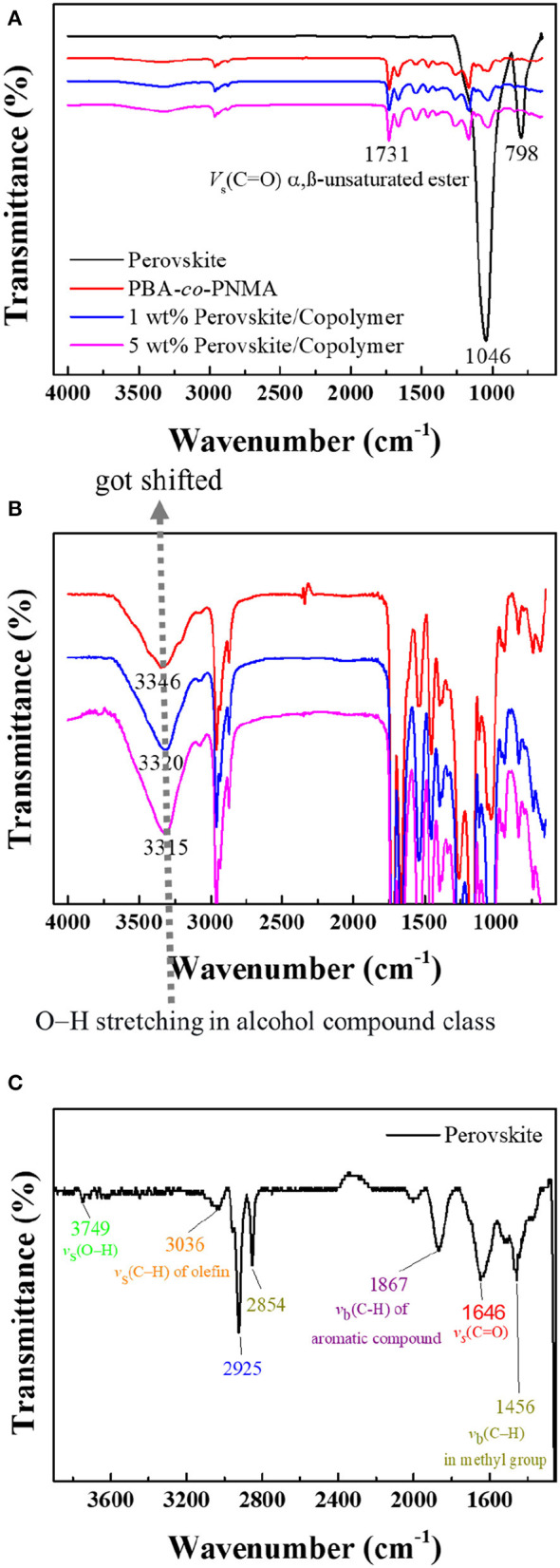
**(A)** FTIR analysis result of pristine CsPbBr_3_ (black line), PBA-*co*-PNMA (red line), and CsPbBr_3_/PBA-*co*-PNMA composites containing 1 wt % (blue line) and 5 wt % (pink line) of CsPbBr_3_. FTIR spectra enlargement of: **(B)** PBA-*co*-PNMA and CsPbBr_3_/PBA-*co*-PNMA composite with different loading ratios of CsPbBr_3_, **(C)** pristine CsPbBr_3_.

Fourier-transform infrared (FTIR) analysis was also performed to investigate the interaction of the CsPbBr_3_ with PBA-*co*-PNMA. Figure 2A shows the FTIR spectra of three thin-film materials spin-coated onto silica wafer (these materials consist of CsPbBr_3_, PBA-*co*-PNMA, and CsPbBr_3_/PBA-*co*-PNMA composite). The peaks that should have indicated the existence of perovskite in composite films were difficult to detect with the FTIR spectrum (including OA ligands) due to the possibility of very low concentration and further being enveloped in a random copolymer matrix. On closer inspection, O–H stretching from the NMA segment shifted with increasing perovskite concentration in random copolymer (Figure 2B). These results can be interpreted as an indication that the embedding of CsPbBr_3_ in random copolymer influences the formation of hydrogen bonds between random copolymer chains, which is crucial for maintaining the self-healing ability of PBA-*co*-PNMA. In CsPbBr_3_ the bands around 675–1,000 cm^−1^ can be assigned to *v*_*b*_(C–H). The peak at 798 cm^−1^ reveals the presence of –(CH_2_)_n_- in saturated long-chain hydrocarbons. The peak in the range of 2,840–2,950 cm^−1^ shows CH_2_ and CH_3_ symmetric and asymmetric stretching vibrations. The other peaks at 1,456, 1,646, 1,867, 3,036, and 3,749 cm^−1^ are ascribed to the *v*_*b*_(C–H) in methyl group, *v*_*b*_(C–H) of an aromatic compound, *v*_*s*_(C–H) of methylene, *v*_*s*_(C–H) of olefin, and *v*_*s*_(O–H), respectively (Figure 2C). All of the aforementioned peaks are similar to those described in other research (Pan et al., 2016; Yan et al., 2019).

**Figure 3:**
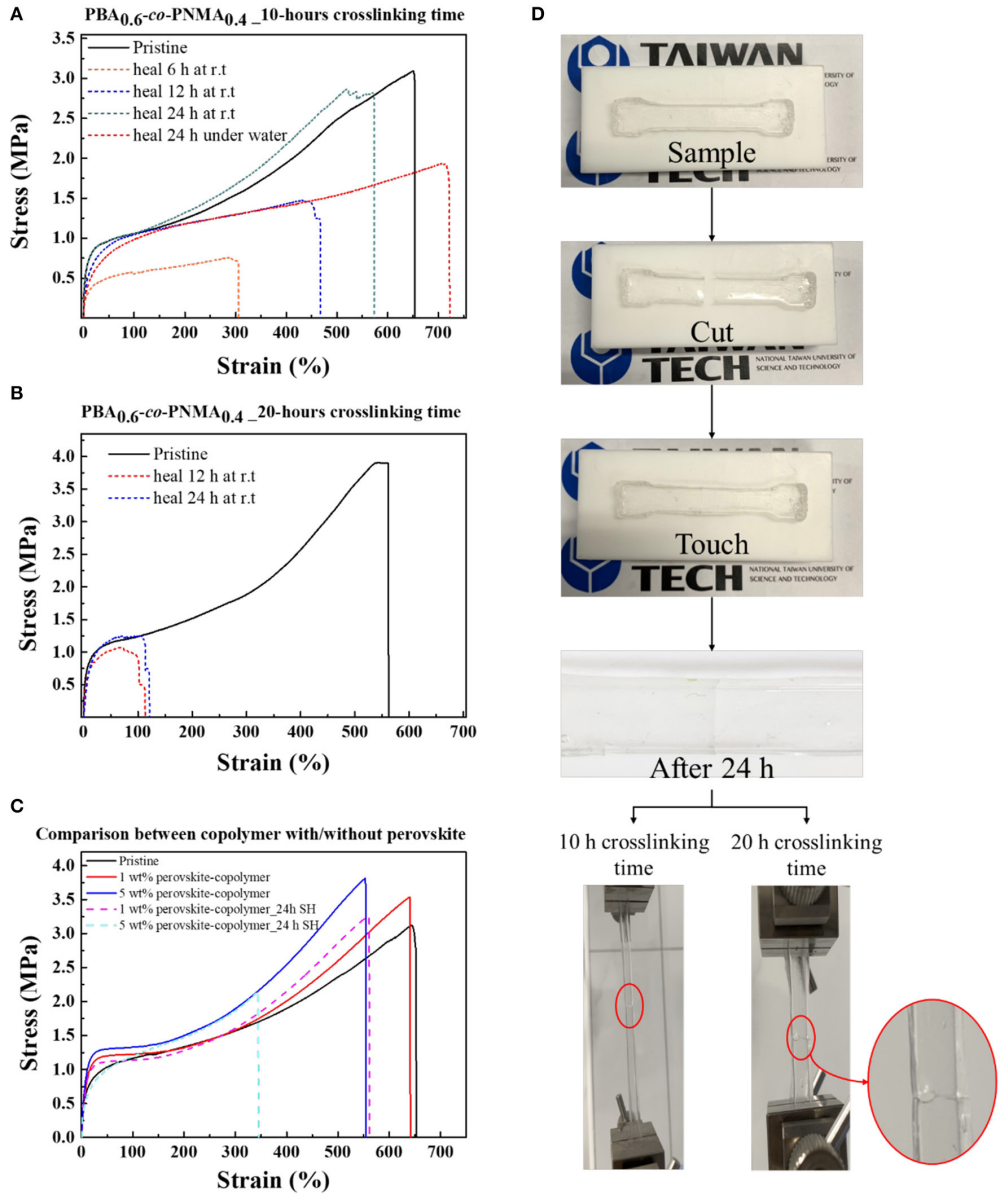
Stress-strain curves of PBA-*co*-PNMA film before self-healing (solid line) and after self-healing (dashes line) test after crosslinking: **(A)** 10 h, **(B)** 20 h. **(C)** Stress-strain curves of PBA-*co*-PNMA with/without perovskite, equipped with self-healing behavior of CsPbBr_3_/PBA-*co*-PNMA composite. **(D)** Photograph demonstrating the self-healing test preparation, using a dog-bone shaped specimen and the stretching behavior of PBA-*co*-PNMA at two different crosslinking times (10 and 20 h) after 24 h of self-healing.

As a consequence of the reversible nature of hydrogen bonding cross-linkages from the NMA segment, the elastic PBA-*co*-PNMA was endowed with excellent autonomous healing capability at room temperature. The mechanical and self-healing properties of the random copolymer were evaluated using a universal testing machine as explained in the section of this paper on the Experiment. Figure 3A displays the stress-strain plots of the pristine (solid line) and healed (dashes line) PBA-*co*-PNMA film after 10-hours of crosslinking time. The success of healing can be verified by the value of all mechanical parameters demonstrated by the stress-strain curve (Young's modulus, toughness, maximum tensile strength, and maximum strain at break), which is nearly coincident to the stress-strain curve before the sample was cut. Remarkably, the mechanical performance of PBA-*co*-PNMA could recover the yield stress and the toughness, even when it was cut into two pieces (Supplementary Table 1). The pristine copolymer exhibits maximum stress of 3.12 MPa and a maximum strain of 653%. Meanwhile, the damaged samples after healing for 6, 12, and 24 h were observed to be restored to 0.76, 1.48, and 2.87 MPa of the maximum stress, and 306, 467, and 573% of the maximum strain, respectively. The self-healing efficiency of damaged samples increases with the extension of healing times. Self-healing efficiency for each sample with different healing times of 6, 12, and 24 h reached 16, 47, and 86%, respectively. On the other hand, Figure 3B shows that the increased maximum stress reaches 3.9 MPa but that the stretching ability decreases to 560% of PBA-*co*-PNMA at 20 h of crosslinking time compared with PBA-*co*-PNMA at 10 h of crosslinking time. Thus, the mechanical properties of PBA-*co*-PNMA are crosslinking time dependent, since NMA plays an important role in providing crosslinking function induced by the thermal reaction, where the initial reaction formed bis (methylene ether) by loss of water and then formed methylene bridge by the loss of formaldehyde to achieve a strong bond structure (Krishnan et al., 2003). As expected, by increasing the crosslinking time, this random copolymer presents plastic behavior, leading to strain hardening and decreasing its ability to self-heal. PBA-*co*-PNMA with 20 h of crosslinking time (dashed line) shows poor self-healing ability where the self-healing efficiency at 24 h only reaches 11.0%.

Intriguingly, the random copolymer film can heal even when being immersed in deionized water. To demonstrate its self-healing capability underwater, a random copolymer was cut into two pieces and the broken parts were gently reconnected in water. After 24 h of self-healing underwater, the maximum strain of the random copolymer was increased but the Young's Modulus and the toughness decreased, as shown in Figure 3A (red dash line). This may be due to the presence of hydrophobic material, BA, as a dominant protector located on the outer surface of the random copolymer film, able to resist or prevent water ions from attacking the NMA group on the cracked surface. We observed no swelling behavior of the random copolymer during the self-healing test, which might further prove the hydrophobicity of BA dominated the random copolymer behavior underwater. Instead, the water molecule may attack the C=O group on the BA segment, causing an increase in ionic mobility in the random copolymer. In short, the random copolymer becomes softer and flexible in water.

The influence of CsPbBr_3_ on the mechanical properties of the random copolymer was studied, as shown in Figure 3C. The embedding of CsPbBr_3_ in the random copolymer system significantly improved the mechanical strength, which exhibits greater maximum tensile stress and higher Young's modulus, as the concentration of CsPbBr_3_ increased from 1 to 5 wt%. The maximum stress of the random copolymer and CsPbBr_3_/PBA-*co*-PNMA composite with 1 and 5 wt% CsPbBr_3_ are 3.12, 3.54, and 3.81 MPa, respectively. Considering the outstanding mechanical properties that resulted from embedding CsPbBr_3_ into the copolymer system, the self-healing ability of 1 and 5 wt% CsPbBr_3_/PBA-*co*-PNMA was also evaluated. The self-healing efficiency of 1 and 5 wt% composites are 80.8 and 42.4%, respectively. The reduction in self-healing efficiency could be due to the interactions between CsPbBr_3_ with dynamic hydrogen bonds in the NMA segment. The possibility of this was proved through FTIR analysis (Figure 2B). A photograph of copolymer film preparation for the self-healing test is illustrated in Figure 3D. The notch on the copolymer film almost disappeared after 24 h of self-healing at room temperature, but an indistinct healed scar was still visible.

**Figure 4:**
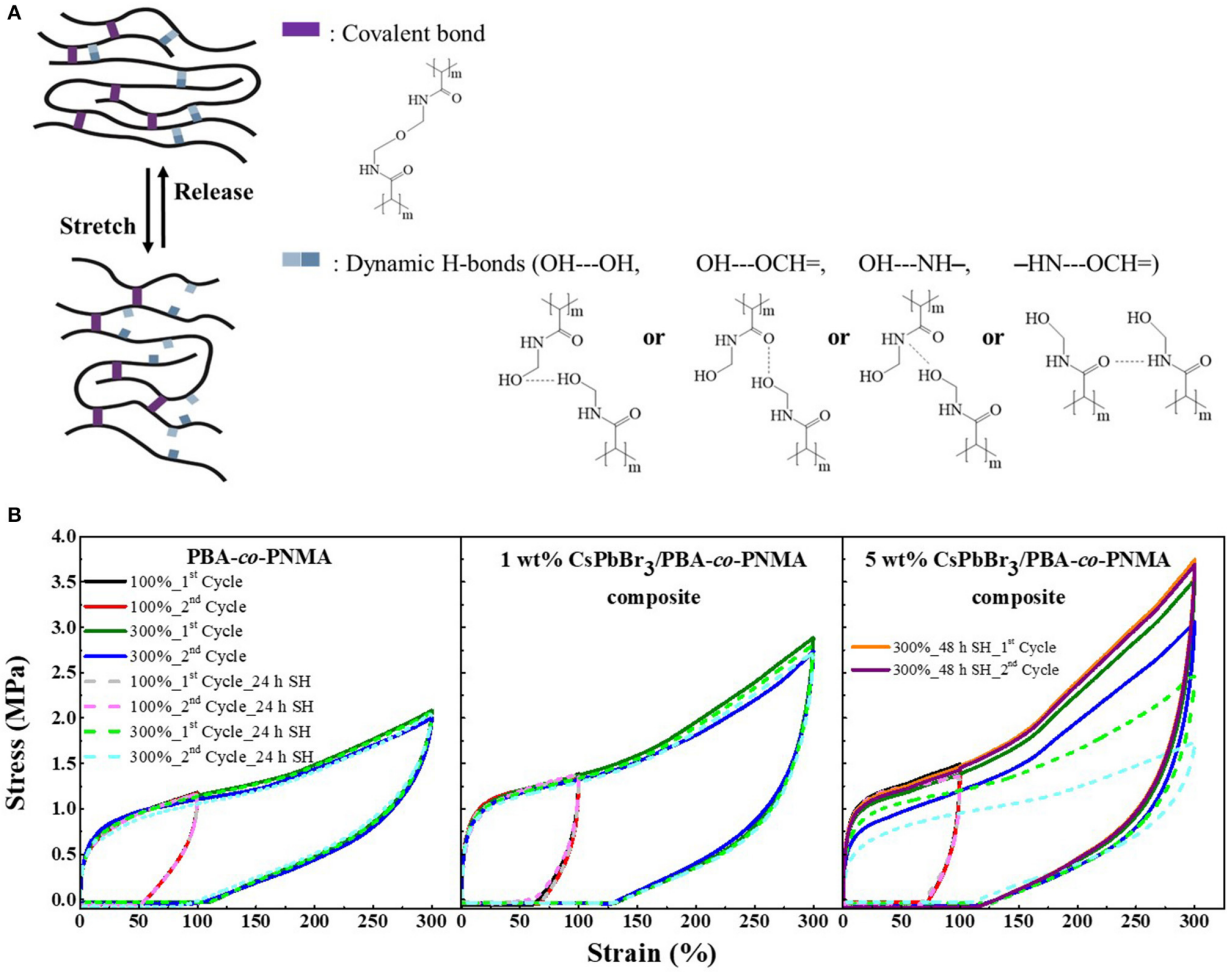
**(A)** Stretch-release simulation of PBA-*co*-PNMA during the cycle test. **(B)** Cyclic tensile curve of PBA-*co*-PNMA, 1 wt% CsPbBr_3_/PBA-*co*-PNMA composite, and 5 wt% CsPbBr_3_/PBA-*co*-PNMA composite subjected to loading-unloading test at 100 and 300% strain; before (solid line) and after self-healing (dash line). The recovery time between two consecutive loading cycles is 5 min. Notably, the two separate time cycle tests (of 5 wt% composite after 48 h self-healing) overlapped with the initial cycle test of 300% strain.

Apart from Visual Observation and Oscillatory Rheology methods used to characterize the self-healing behavior of healed-materials, cycle tensile testing is also a principal method of evaluating the self-recovery as well as the self-healing ability of an elastomer (Strandman and Zhu, 2016). The good elasticity of PBA-*co*-PNMA is attributed to the correlation between covalent and dynamic H-bonds that link each NMA segment through chemical and physical cross-linking reaction. The covalent cross-links strengthen and preserve the polymer networks, while the reversible H-bonds can dynamically cleave and reform during the loading-unloading test. As illustrated in Figure 4A, reversible H-bonds consisting of OH—OH, OH—NH, OH—=OC, and NH—=OC act as sacrificial bonds that are ruptured upon loading to dissipate energy, and reformed after unloading to restore structures and mechanical properties. Meanwhile, the covalent bonds are kept intact before and after stretching. The combination of the covalent network and sacrificial bonds in a random copolymer system was expected to endow extraordinary recoverability with high Young's modulus and maximum stress.

Figure 4B compares the stress-strain data of the pristine copolymer and CsPbBr_3_/PBA-*co*-PNMA composite containing 1 and 5 wt% CsPbBr_3_ in two different conditions [i.e., before (solid line) and after the self-healing (dash line) process]. In the first condition, cycle tensile tests of PBA-*co*-PNMA and CsPbBr_3_/PBA-*co*-PNMA composite containing 1 and 5 wt% CsPbBr_3_ were performed with increasing maximum strain from 100 to 300% and with a short rest-time of 5 min between cycles. After the first tensile cycle with a small strain (100%), the unbroken dynamic H-bonds encouraged the networks back to their original state. The specimen-films displayed excellent self-recovery, in which only a short amount of time (5 min) was required to fully return to their original dimension, successfully restoring the original stress-strain performance, as demonstrated through the second cycle test. In a larger strain (300%), PBA-*co*-PNMA and 1 wt% CsPbBr_3_/PBA-*co*-PNMA can still return to its original form perfectly after 5 min of relaxation, and only a slight reduction in mechanical strength performance was detected. Meanwhile, the mechanical strength of 5 wt% CsPbBr_3_/PBA-*co*-PNMA was dramatically reduced after the first cycle test at 300% strain, due to the reformation of broken sacrificial dynamic bonds, which did not have enough time to restore to their original stress-strain performance during the limited timeframe. The second specimen condition was conducted after self-healing for 24 h at room temperature. With the same cycle test procedures, each healed-specimen showed complete recovery after loading-unloading for two times at a low strain region (100%). The stress-strain curve was almost identical to the first condition. However, a distinguishable reduction in both Young's modulus and toughness was observed in 5 wt% composite in the loading-unloading test at 300% strain, due to imperfect self-healing within 24 h. This behavior is attributed to the interactions between the perovskite and the copolymer, indicating less dynamic H-bond interaction between each NMA segments in the 5 wt% composite system. The tensional strength of 5 wt% composite can be restored by prolonging the rest periods or healing time (48 h), as evidenced by an overlap of cycle curves (Supplementary Figure 1). By focusing on the cycle test of each specimen film before and after self-healing at 300% strain, it was revealed that the specimen not only successfully self-healed but that it was also able to restore the mechanical performance to its original condition. PBA-*co*-PNMA presents an impressive balance between elasticity, mechanical strength, and an outstanding and dynamic self-healing ability at room temperature. Even after embedding the CsPbBr_3_ into the copolymer system, the admirable mechanical properties, elasticity, and self-healing capability were maintained. This unique copolymer can be widely developed by combining various types of perovskite or other nanomaterials and is expected to have promising potential applications as a protective film for optical lenses, flexible display screens, electronic skin, amongst other applications.

**Figure 5:**
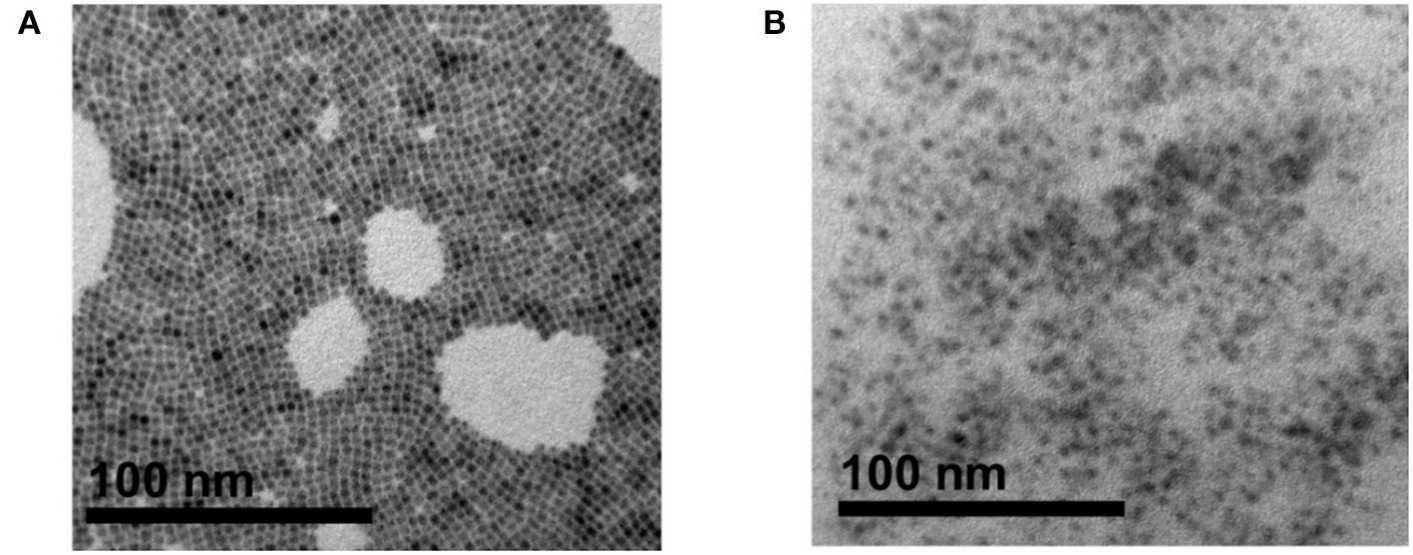
Transmission electron microscopy (TEM) image of: **(A)** pristine CsPbBr_3_ crystal, **(B)** CsPbBr_3_/PBA-*co*-PNMA composite. Inset for each image are the HRTEM image result, prepared by drop-cast on a copper grid.

To investigate the morphology and size distributions of CsPbBr_3_ in the random copolymer, the perovskite materials were characterized by transmission electron microscopy (TEM). The TEM images of pristine CsPbBr_3_, Figure 5A, show that the CsPbBr_3_ possesses a cubical perovskite structure, though few aggregations are still observed. However, after introducing the perovskite into a copolymer matrix, aggregation can be prevented, as depicted in Figure 5B in which the CsPbBr_3_ are well-dispersed in random copolymer without obvious aggregation.

**Figure 6:**
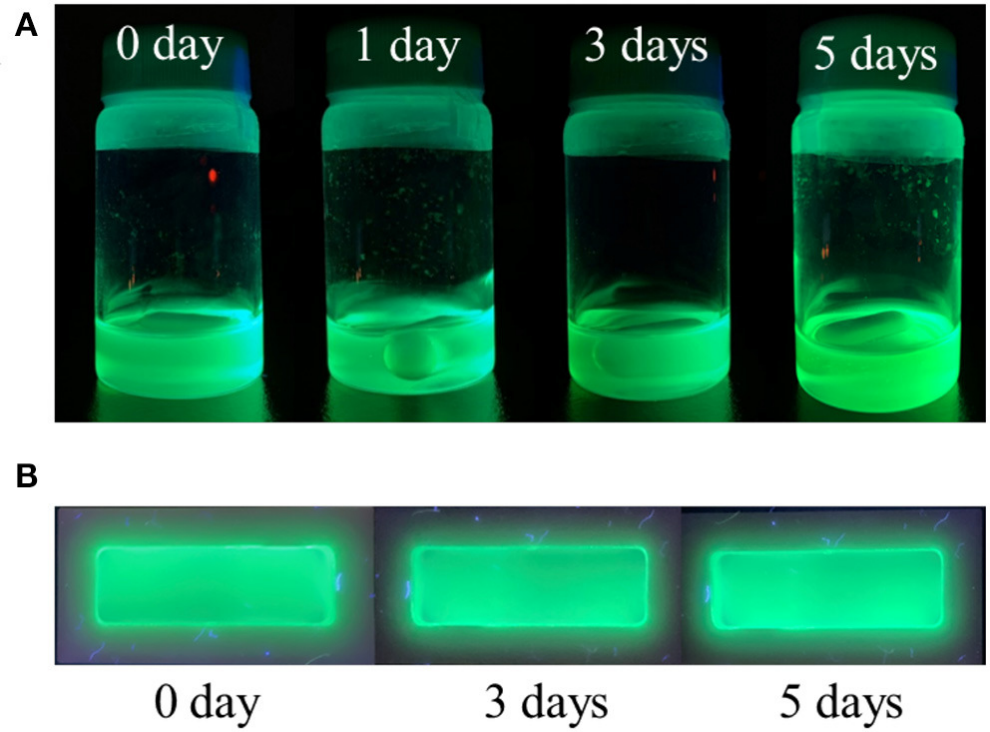
The photograph of 5 wt% composite (CsPbBr_3_/PBA-*co*-PNMA-toluene solvent) under 365 nm UV Light. In: **(A)** solution form, **(B)** film form after 10-h crosslinking reaction.

**Figure 7:**
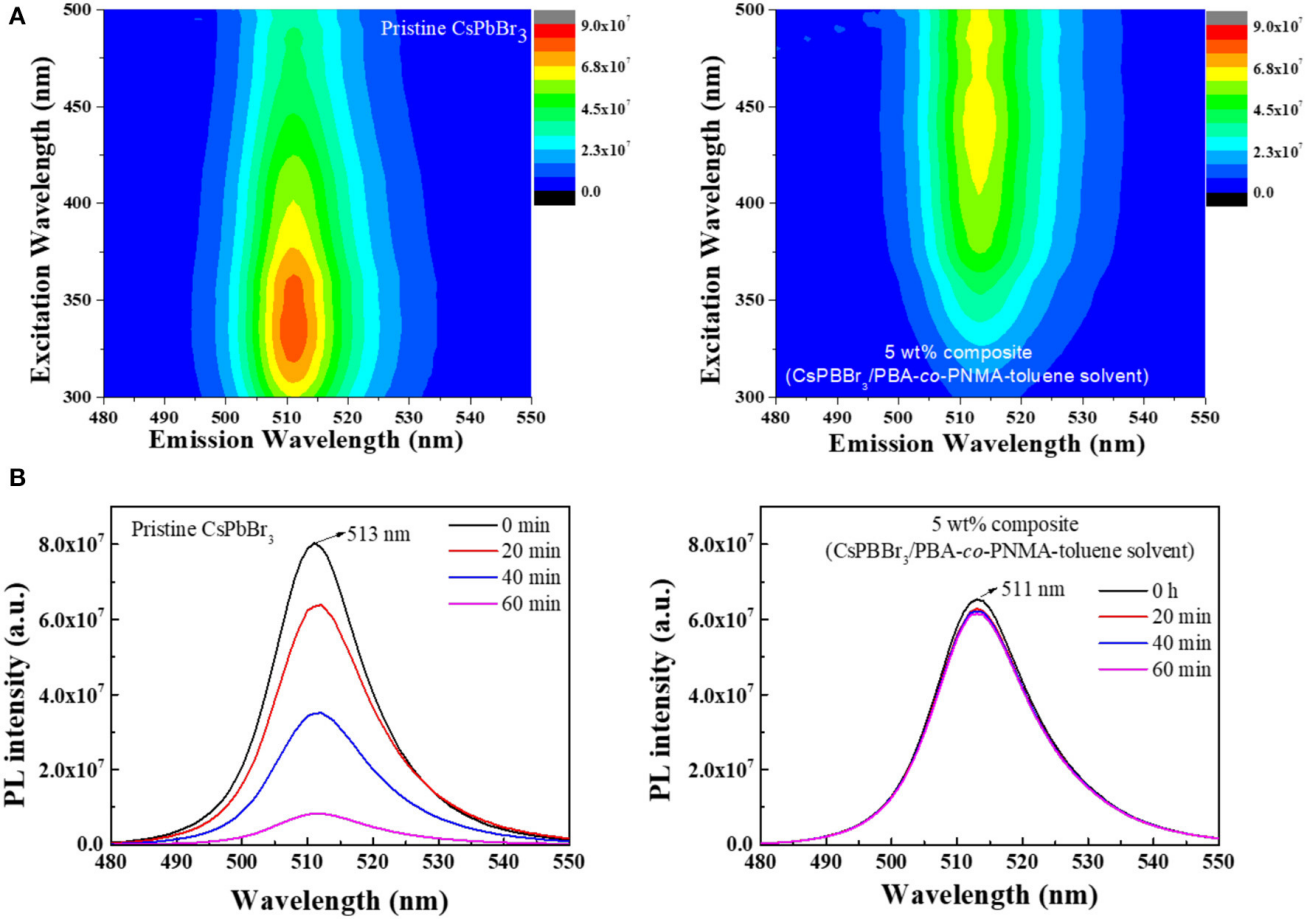
**(A)** The PLE mapping of CsPBBr_3_ and CsPbBr_3_/PBA-*co*-PNMA composite at initial condition. **(B)** The PL stability of CsPBBr_3_ and 5 wt% CsPbBr_3_/PBA-*co*-PNMA composite in liquid form after exposure to air at different times.

Practical applications of pure CsPbBr_3_ are severely limited because it exhibits strong green emission and poor stability in open-air, which are associated with the degradation of CsPbBr_3_ upon exposure to water or water vapor (Habisreutinger et al., 2014; Shi et al., 2017). This instability in open-air can be improved, potentially for long periods, by embedding CsPbBr_3_ into a copolymer system. In this study, 5 wt% composite was chosen to represent the composite emission under UV light. Figures 6A,B respectively visualize the strong green emission of 5 wt% CsPbBr_3_/PBA-*co*-PNMA composite in solution and film states under 365 nm UV illumination. No emission decay was observed even after 5 days of exposure to open air. The stability was further investigated analytically by PL measurement (Figure 7). Figure 7A shows the excitation-independent PLE mapping of pristine CsPbBr_3_ and 5 wt% composite in solution form. Even though there is a red-shift behavior in the maximum excitation wavelength (from 335 to 440 nm) after perovskite incorporation into the copolymer matrix, the corresponding maximum emission wavelength is still maintained at 511 nm.

Figure 7B indicates changes in PL intensity over time. The PL intensity of pristine CsPbBr_3_ was dispersed in toluene in the initial 20 min to the last 1 hour and decreased significantly when it came into direct contact with air. After 1 hur, the PL intensity decayed to 89.7% of the initial value and then continued to decrease until the perovskite emission decayed completely. In contrast to perovskite, the PL intensity of 5 wt% composite in solution form in the initial 20 min only decreased to 6% of the initial value and then remained stable for the remaining 1 h. The first decrease of PL intensity could be due to the degradation of CsPbBr_3_ located on the surface of the solution. In this situation, the hydrophobic nature of BA plays an important role in preventing further decomposition of CsPbBr_3_, in which the PL intensity remained constant. The lower PL intensity exhibited by the CsPbBr_3_/PBA-*co*-PNMA composite solution compared with the pure perovskite solution (Figure 7B) can be attributed to the 2 days blending process time. Perovskite decomposition was inevitable due to contact with the air, even though the blending was conducted in a closed system. Our strategy takes this into account, as it improves the stability of CsPbBr_3_ in water.

**Figure 8:**
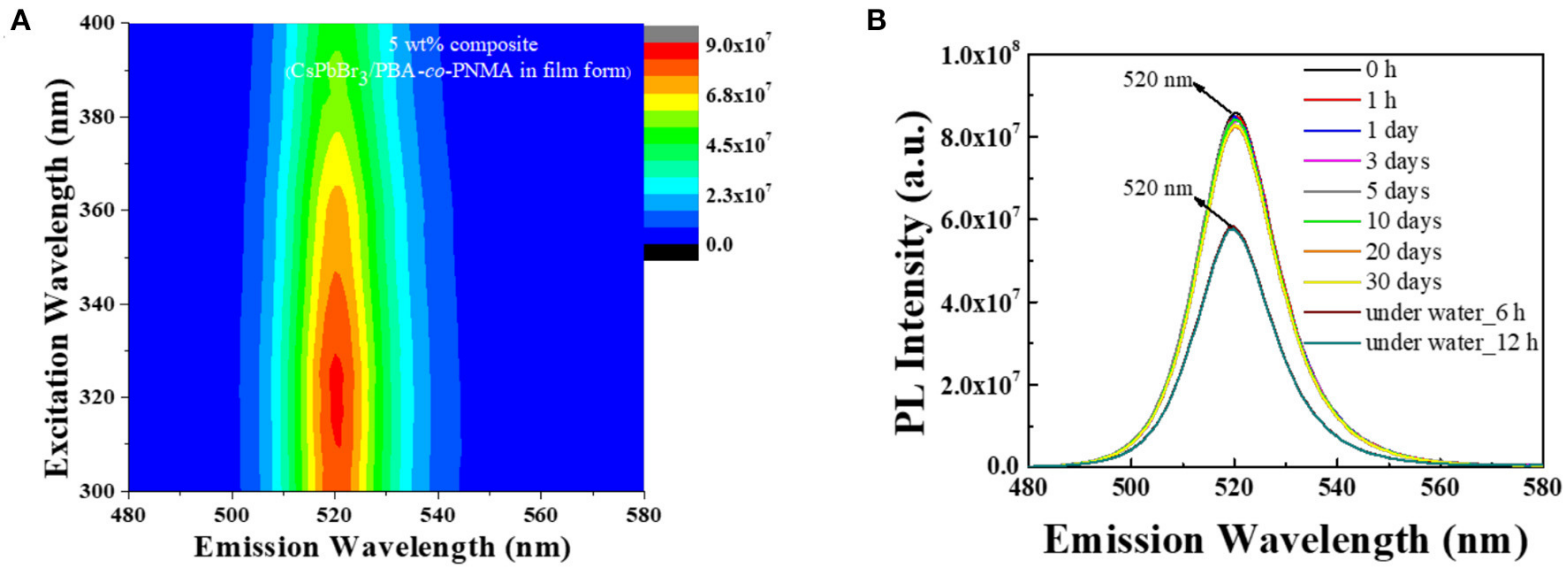
**(A)** The PLE mapping at the initial time (*t* = 0 h), and **(B)** PL stability of 5 wt% composite (CsPbBr_3_/PBA-*co*-PNMA) film after 10 h crosslinking in open-air condition and immersed in water for a certain time.

The embedding of CsPbBr_3_ in random copolymers affects the formation of hydrogen bonds between copolymer chains, which can cause changes to mechanical and luminescence properties. Furthermore, when that CsPbBr_3_/PBA-*co*-PNMA composite is physically cross-linked, the polymer molecule will be more stable, since the free hydroxyl groups are decreased, reducing the hydrophilicity of NMA. In addition, the perovskite molecules will be locked inside the polymer host so that there will be no particle agglomeration (like in the liquid phase), as shown in Figure 5. Figure 8A shows the PLE emission of the composite film and Figure 8B shows the corresponding PL stability after exposure to air, at different times. Instead of decreasing with longer air exposure like in the solution case, the PL intensities of the composite film remained largely constant, as described in Figure 8B. This result suggests that most of the perovskite have been successfully protected by their polymer host, preventing decomposition when the film is exposed to the surrounding air. Moreover, in water immersion, the PL intensities remained constant, even with longer immersion time. Despite losing 32% of its intensity, a relatively strong PL was still exhibited by the PBA-*co*-PNMA/CsPbBr_3_ film, as the hydrophobic BA provided water protection to the CsPbBr_3_.

After cross-linking at 90°C, there was no apparent perovskite decomposition since the perovskite used in this study is resistant to high temperatures, as shown in Supplementary Figure 2. The GIXRD images show that CsPbBr_3_ can withstand 150°C without decomposition. The d-spacing of CsPbBr_3_ is 0.5, as indicated above. Owing to the multifunctionality of each material used in this research, the resulting composite can be an example of combining 3 types of materials with their own advantages that can cover each of their own weakness.

## Conclusion

In summary, this study demonstrated a convenient and simple strategy for the preparation of inorganic halide perovskite CsPbBr_3_–random copolymer composite. The as-prepared CsPbBr_3_/PBA-*co*-PNMA composite not only showed high stability against water and heat but also demonstrated an ability to stretch and undertake autonomous healing. More interestingly, after CsPbBr_3_/PBA-*co*-PNMA composite film crosslinking for 10 h, the photoluminescence intensity of CsPbBr_3_ was further enhanced because there is no perovskite aggregation and decomposition upon contact with the surrounding air. Based on the above results, the composite has great potential in enabling next-generation smart optical devices to have the ability to self-heal in any conditions (for example, in ambient conditions, high temperatures, or even underwater).

## Data Availability Statement

All datasets generated for this study are included in the article/Supplementary Material.

## Author Contributions

LL and Y-CC conceived the idea and designed the experiments. LL, YF, and CA synthesized the polymers and CsPbBr_3_ and carried out the characterization. A student of Professor Chen prepared the CsPbBr_3_ under the supervision of L-YC, LL, CA, Y-TL, and A-NA-D performed the stretching, photophysical, and morphological characterization. LL, A-NA-D, and Y-CC prepared the paper. All authors read and approved the final manuscript.

## Conflict of Interest

The authors declare that the research was conducted in the absence of any commercial or financial relationships that could be construed as a potential conflict of interest.
