# Supplementary material for: Improving the Lifetime of CsPbBr3 Perovskite in Water Using Self-Healing and Transparent Elastic Polymer Matrix
Source: Front Chem. 2020 Oct 6;8:766. doi: 10.3389/fchem.2020.00766 (PMC7573164; doi:10.3389/fchem.2020.00766)
Supplement: Supplementary file 1 [file Data_Sheet_1.docx]

**Improving the lifetime of CsPbBr_3_ Perovskite in Water using Self-Healing and Transparent Elastic Polymer Matrix**

Livy Laysandra^1†^, Yong Jie Fan, ^1†^ Cecilia Adena^1†^, Yen-Ting Lee^1^, Ai-Nhan Au-Duong^1^,

Liang-Yih Chen^1^, Yu-Cheng Chiu^1,2^*

*^1^Department of Chemical Engineering, National Taiwan University of Science and Technology, Taipei 10607, Taiwan.*

*^2^Graduate Institute of Applied Science and Technology, National Taiwan University of Science and Technology, Taipei, Taiwan*

* Author to whom all correspondence should be addressed. Email: [ycchiu@mail.ntust.edu.tw](mailto:ycchiu@mail.ntust.edu.tw)


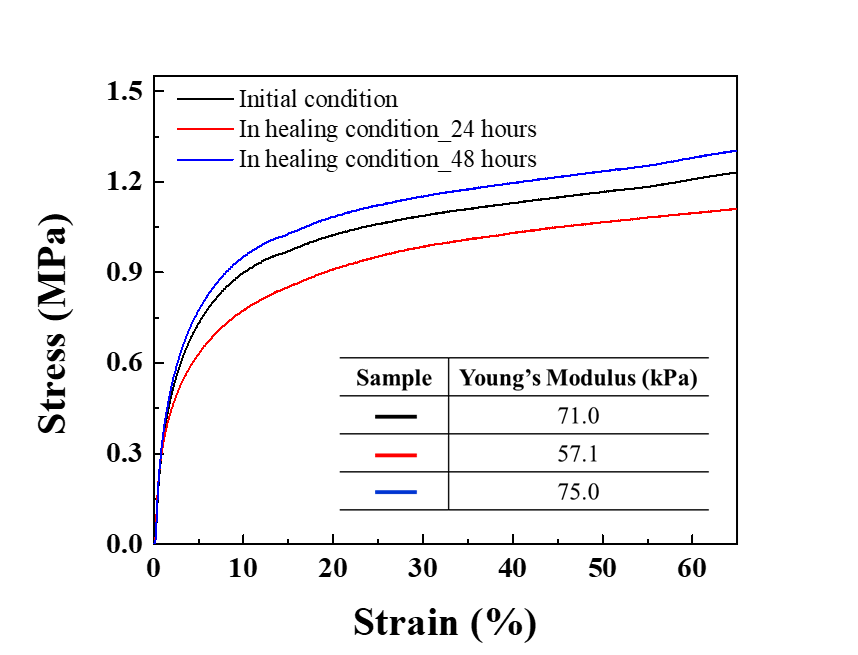


**Supplementary Figure S1 |** Enlargement from the first cycle test of 5 wt% CsPbBr_3_/PBA-*co*-PNMA in three different conditions showed in **Figure 4B** representing a region of strain from 0 to 65% and the inset table showing the mechanical parameter. The Young’s Modulus was calculated from stress at a small strain (≤ 10%)

**
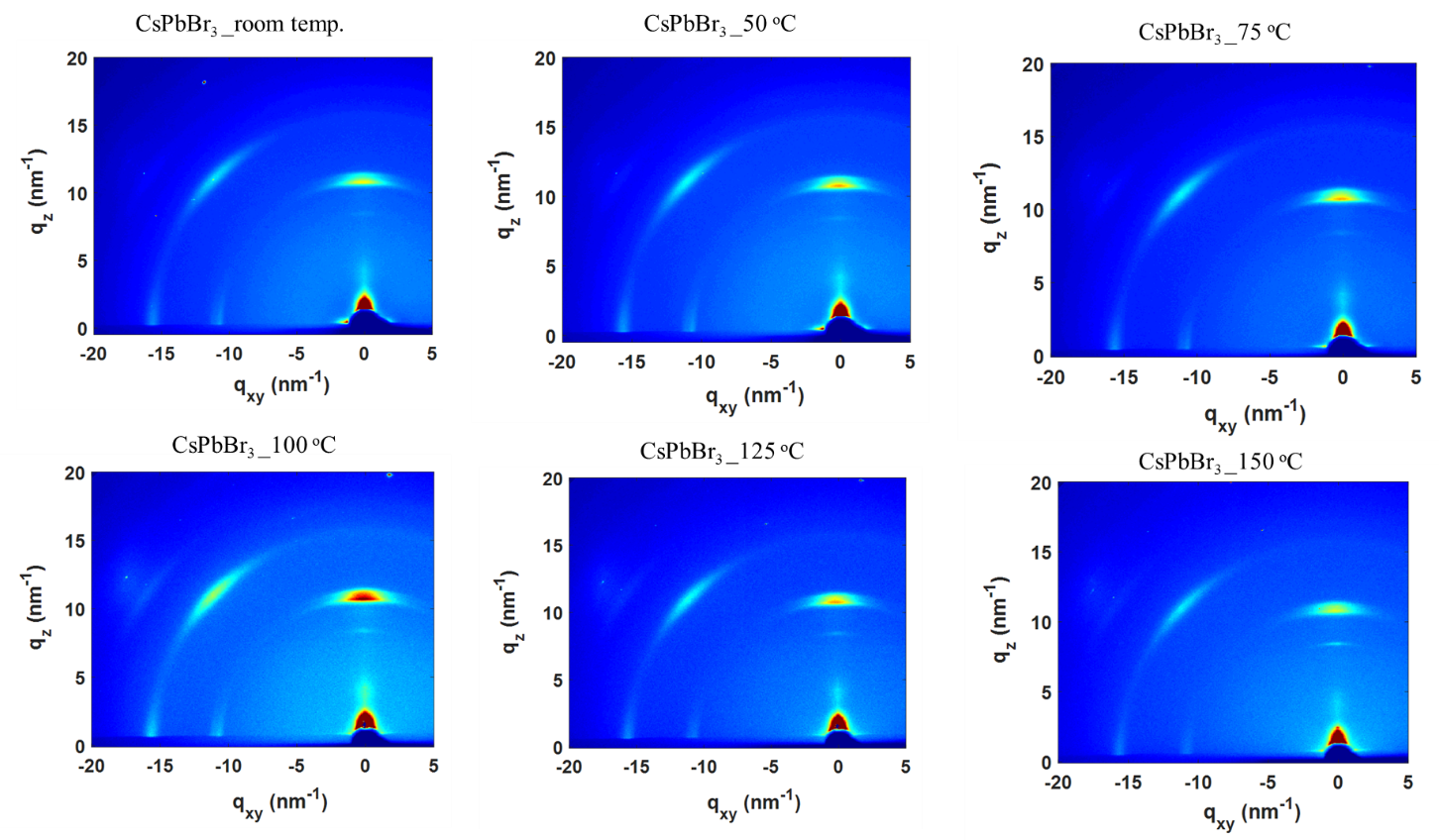
**

**Supplementary Figure S2 |** Grazing incidence X-ray Diffraction measurement results of pristine CsPbBr_3_ at various temperature from room temperature until 150°C

**Table S1.** Mechanical properties of PBA-*co*-PNMA at 10-hours crosslinking time before and after self-healing in different healing time under ambient condition (measured at a stress-strain speed of 50 mm min^−1^).

| Specimen | Maximum stress (MPa) | Maximum strain (%) | Young’s Modulus^a^ (kPa) | Toughness **^b^** (MJ/m^3^) |
| --- | --- | --- | --- | --- |
| Pristine | 3.12±0.02 | 653±0.81 | 51±0.21 | 11.69±0.002 |
| 6 hours healing time | 0.76±0.05 | 306±3.36 | 24±3.50 | 1.84±0.15 |
| 12 hours healing time | 1.48±0.13 | 467±2.20 | 42±4.96 | 5.50±0.04 |
| 24 hours healing time | 2.87±0.20 | 573±8.74 | 51±3.46 | 10.02±0.41 |

^a^Calculated from stress at a small strain (≤ 10%). ^b^Estimated by the area under stress-strain curve until fracture point.
